# Vocabulary knowledge as a reliable proxy of cognitive reserve in multiple sclerosis: a validation study

**DOI:** 10.1007/s10072-024-07388-w

**Published:** 2024-02-28

**Authors:** Gianpaolo Maggi, Manuela Altieri, Mario Risi, Valentina Rippa, Riccardo Maria Borgo, Rosaria Sacco, Daniela Buonanno, Alessandro D’Ambrosio, Alvino Bisecco, Gabriella Santangelo, Antonio Gallo

**Affiliations:** 1 Department of Advanced Medical and Surgical Sciences, University of Campania “Luigi Vanvitelli”, Naples, Italy; 2 Department of Neurology, Neurocenter of Southern Switzerland (NSI), Regional Hospital of Lugano, Ente Ospedaliero Cantonale, Lugano, Switzerland; 3 Department of Psychology, University of Campania “Luigi Vanvitelli”, Caserta, Italy

**Keywords:** Cognitive reserve, Multiple sclerosis, Vocabulary knowledge, Validation, Reliability

## Abstract

**Introduction:**

The present study aimed to explore the suitability of the vocabulary knowledge (VOC) test as an accurate and reliable proxy of cognitive reserve (CR) by evaluating its psychometric properties and discrimination accuracy compared with other CR measures in multiple sclerosis (MS).

**Methods:**

Sixty-eight consecutive people with multiple sclerosis (pwMS), followed at our MS outpatient clinic, completed a clinical evaluation and neuropsychological assessment including: VOC, Brief Repeatable Battery of Neuropsychological Tests (BRB-N), Cognitive Reserve Index Questionnaire (CRIq), Beck Depression Inventory-II, and State-Trait Anxiety Inventory. Reliability, convergent and divergent validity, and discrimination accuracy of the VOC were assessed using educational level as reference standard. The possible effects of sociodemographic and clinical factors on VOC and their role in predicting global cognitive status were also explored.

**Results:**

VOC demonstrated good internal consistency (Cronbach’s *α* = 0.894) and adequate construct validity. It showed an acceptable level of discrimination between pwMS with high and low CR, comparable to the CRIq score. Education strongly affected VOC scores, which in turn were independent of MS features. VOC emerged as an independent predictor of global cognitive status together with MS-related disability.

**Conclusion:**

We demonstrated the validity of VOC as a reliable CR measure in pwMS. Thus, CR may also be estimated using fixed objective measures, independent of brain pathology and clinical features. Early CR estimation may help clinicians identify pwMS at a higher risk of cognitive decline and plan strict neuropsychological monitoring and cognitive interventions.

## Introduction

Cognitive changes in multiple sclerosis (MS) are estimated to affect 40-70% of patients because of chronic and multifocal MS-related central nervous system (CNS) damage [1]. Cognitive impairment is one of the most disabling manifestations in people with MS (pwMS), with detrimental consequences for individuals’ quality of life, performance, and achievements at study/work and in everyday activities [2]. However, extreme variability has been observed in MS-related cognitive deficits, with pwMS being able to withstand a considerable disease burden. This cognitive-neuropathologic dissociation is not exclusive to MS; indeed, it has also been described in Alzheimer’s disease (AD) [3] and other neurological conditions [3, 4]. This mismatch has prompted the elaboration of the concept of a structural and functional brain reserve that may explain individual differences in the delay in time between pathology and clinical expression of cognitive decline [5, 6].

The brain's capacity to cope with age-related brain changes and pathologic damage has been defined as cognitive reserve (CR), which recruits protective/compensatory mechanisms associated with cognitive abilities built up over the course of life [3, 4]. Numerous operationalizations of the latent concept of CR have attempted to quantify the level of reserve by proposing educational or occupational attainment, together with engagement in leisure and social activities, as the most influential proxy of CR [3, 7]. However, in clinical practice, CR evaluation is commonly limited to the highest attained educational level; this view presents some limitations because the educational level is strongly influenced by socioeconomic and cultural factors [5, 8] in terms of possibilities and resources needed to afford higher educational attainment and subsequent leading occupational position as well as an enriching environment [9], which results in being too simplistic to capture the dynamic and multidimensional nature of CR across life [10].

Multi-item surveys, including the assessment of socio-behavioral factors at different life stages, have been developed to overcome these limitations [11–13]; however, most of these do not consider crucial CR contributors, such as educational attainment and occupational history. Of these, the Cognitive Reserve Index Questionnaire (CRIq) has proven to be an efficient and reliable tool for measuring CR by integrating information related to educational level and occupational status with items assessing activities carried out throughout adulthood and current lifestyle (e.g., time spent performing cognitively, socially, and/or physically stimulating leisure activities or hobbies) [14].

Within this framework, lifetime intellectual enrichment represents a crucial source of CR and may be estimated by objective measures such as vocabulary knowledge (VOC), a test validated and standardized in several languages as a subtest of the Wechsler Adult Intelligence Scale (WAIS), which evaluates semantic knowledge acquired through enriching life activities, such as educational attainment, occupation, and frequent reading [15, 16]. Some studies revealed that higher CR assessed by the VOC was associated with better performance on cognitive tests in MS, independent of brain pathology and clinical features [17, 18], moderating the impact of lesion load on cognition in MS [19, 20]. However, to the best of our knowledge, no study has assessed the reliability and discrimination accuracy of this tool as a proxy for CR in pwMS. Therefore, the present study aimed to (i) examine the validity and reliability of the VOC as a suitable proxy of the CR and (ii) evaluate its discrimination accuracy compared to other CR measures, such as educational level and CRIq scale, in an Italian cohort of pwMS.

## Materials and methods

### Participants

Consecutive pwMS followed at the MS outpatient clinic of the Division of Neurology of the University of Campania “Luigi Vanvitelli,” in Naples (Italy) were screened and enrolled in the study. Participants were included in the study if they met the following inclusion criteria: (i) a diagnosis of MS according to the revised McDonald criteria [21], (ii) absence of psychiatric comorbidities and major neurocognitive disorders according to DSM-5, and (iii) absence of clinical relapse and use of corticosteroids or other drugs affecting cognitive functions within 3 months of the evaluation.

Demographic (i.e., age, sex, and years of schooling) and clinical aspects, such as disease duration and the Expanded Disability Status Scale (EDSS), were recorded by a neurologist with expertise in MS.

All participants signed an informed consent form to participate in the study, which was approved by the Local Ethics Committee and was performed in accordance with the ethical standards laid down in the 1964 Declaration of Helsinki and its later amendments.

### Neuropsychological assessment

The participants completed the VOC of the WAIS. This test evaluates vocabulary knowledge by asking examinees to explain the meaning of 35 words. VOC is considered an estimate of lifetime intellectual enrichment because it is strongly influenced by enriching life activities (e.g., education, occupation, and reading) [3]. Raw VOC scores were used for the analyses.

All participants underwent the Italian version of Rao’s Brief Repeatable Battery of Neuropsychological Tests (BRB-N) [22]. The BRB-N comprises seven cognitive tests, administered in a fixed order, to assess verbal memory (Selective Reminding Test; SRT), visuospatial memory (10/36 Spatial Recall Test; SPART), attention, working memory, speed of information processing (Paced Auditory Serial Addition Test 3- and 2-s interval versions and Symbol Digit Modality Test; PASAT-3″, PASAT-2″, and SDMT), and semantic verbal fluency (Word List Generation; WLG). In addition, the interference subtask of the Stroop Color Word Interference Test was employed to evaluate inhibitory control [22]. Z-scores were calculated for each sub-score and the overall cognition score was computed by summing all z-scores.

To assess convergent validity, participants completed the CRIq, a multi-item interview widely used in pwMS [23, 24], designed to evaluate CR by combining three different sources: educational attainment, working activity, and engagement in leisure time activities [14]. It comprises three different subscores: The CRI-Education subscore counts the years of formal education plus training and professional courses with a duration of at least six months; the CRI-Working Activity reflects the number of working years based on the cognitive load of the occupation, defined considering intellectual involvement and responsibility; and finally, the CRI-Leisure Time considers all intellectual, social, and leisure activities (e.g., reading books, practicing sports, volunteering, travelling, caring for children/pets) practiced during adulthood. The CRIq total score is the average of the three subscores (standardized and transposed to a scale with mean = 100 and SD = 15), with higher CRIq scores reflecting higher estimated CR.

Finally, the Beck Depression Inventory-II (BDI-II) and State-Trait Anxiety Inventory (STAI-Y), validated in pwMS [25, 26], were employed to assess depressive and anxiety symptoms, respectively. Scores ≥ 19 on the BDI-II indicated the presence of clinically relevant depressive symptoms [25], whereas scores ≥ 64 on the STAI-Y indicated the presence of significant anxiety symptoms [26].

### Statistical analysis

Data quality of data was defined as appropriate in the absence of missing values and by low percentages of floor and ceiling effects, following previous validation studies [27, 28]. Univariate normality was assessed by checking skewness and kurtosis values for the variables of interest; values not exceeding |2| are typically considered indicative of a normal distribution [29–31]. Descriptive variables are then reported accordingly with non-normal variables reported as median and interquartile range and normal variables as mean and standard deviation (SD).

Internal consistency was tested using Cronbach’s *α* coefficient with values ≥ 0.70 considered indicative of acceptable internal consistency [32]. We obtained additional evidence on the reliability and scaling assumptions for each item using Pearson’s item-total correlations and corrected item-total correlations to adjust for inflation errors [27]. Cohen’s conventions (weak, *r* < 0.30; moderate, *r* = 0.30–0.50; strong, *r* > 0.50) were used to interpret the effect sizes.

Convergent validity was assessed by correlations between the VOC and CRIq total scores, whereas divergent validity was evaluated by correlations with the total scores of the BDI-II and STAI-Y. The potential effects of demographic (i.e., age, sex, and educational level) and clinical factors (i.e., EDSS and disease duration) on the VOC score were evaluated using multiple regression analysis.

Receiver operating characteristics (ROC) analysis using education level as the gold standard (years of schooling ≥ 16) was performed to test the accuracy of VOC compared to CRIq in classifying individuals with low and high CR. Intrinsic properties, sensitivity (Se) and specificity (Sp), were determined at the optimal cutoff identified using Youden’s *J* statistic [33].

Multiple regression analysis was carried out to explore the possible effect of VOC on cognition by entering the overall BRB-N composite score as a dependent variable and age, years of schooling, sex, EDSS, disease duration, and vocabulary as predictors controlling for multicollinearity by checking tolerance and variance inflation factor (VIF).

Statistical analyses were performed using IBM SPSS Statistics version 29.

## Results

Sixty-eight (62% males) pwMS were enrolled with a mean age of 41.66 (SD = 13.17) years and an average education of 14.07 (SD = 3.58). Fifty-two (76.5%) pwMS were relapsing–remitting MS (RRMS), three (4.4%) had primary progressive MS (PPMS), and 5 (7.3%) were secondary progressive (SPMS), with a mean EDSS score of 2.60 (SD = 1.77) and an average disease duration of 10.20 years (SD = 9.42) (Table 1). The mean VOC score was 45.32 (SD = 12.51).

**Table 1:** Descriptive statistics on demographic, clinical, and neuropsychological variables

	Mean ± SD
Age (*ys*)	41.66 ± 13.17
Education (*ys*)	14.07 ± 3.58
Sex, *n* (*%*)	M = 26 (38.2%); F = 38 (61.8%)
Disease duration (*ys*)	10.20 ± 9.42
Phenotype	RRMS = 52 (76.5%); PPMS = 3 (4.4%); SPMS = 5 (7.3%)
EDSS	2.60 ± 1.77
VOC	45.32 ± 12.51
BDI-II	10.72 ± 9.02
STAI-Y	74.71 ± 33.36

*SD* standard deviation, *ys* years, *n* number, *M* males, *F* females, *RRMS* relapsing–remitting multiple sclerosis, *PPMS* primary progressive multiple sclerosis, *SPMS* secondary progressive multiple sclerosis, *EDSS* Expanded Disability Status Scale, *VOC* vocabulary knowledge, *BDI-II* Beck Depression Inventory — II, *STAI-Y* State-Trait Anxiety Inventory-Y form

### Reliability

The VOC demonstrated acceptable internal consistency, as indicated by Cronbach’s *α* of 0.894. Most of the items showed an acceptable level of discrimination (items 2, 5, 7, 8, 10–13, 15, 18–20, 22–24, and 26–35; corrected item-total correlations range = 0.319–0.641). Although some items (1, 3, 4, 6, 9, 14, 16, 17, 21, and 25; corrected item-total correlations range =  − 0.017 to 0.288) demonstrated an unsatisfactory level of discrimination (Table 2), these were retained for subsequent analyses to maintain the original structure of the scale.

**Table 2:** Item characteristics of the vocabulary

	Mean ± SD	Item-total correlation	Corrected item-total correlation	Cronbach’s *α* if item removed
*Item 1*	1.87 ± 0.49	0.321	0.286	0.894
*Item 2*	1.74 ± 0.66	0.381	0.334	0.893
*Item 3*	1.96 ± 0.27	0.004	− 0.017	0.896
*Item 4*	1.84 ± 0.41	0.246	0.215	0.894
*Item 5*	1.49 ± 0.68	0.367	0.319	0.893
*Item 6*	1.90 ± 0.35	0.205	0.177	0.895
*Item 7*	1.37 ± 0.69	0.549	0.508	0.890
*Item 8*	1.66 ± 0.66	0.516	0.475	0.891
*Item 9*	1.56 ± 0.63	0.286	0.239	0.894
*Item 10*	1.34 ± 0.94	0.666	0.620	0.888
*Item 11*	1.09 ± 0.75	0.389	0.337	0.893
*Item 12*	0.76 ± 0.71	0.499	0.455	0.891
*Item 13*	1.24 ± 0.81	0.459	0.406	0.892
*Item 14*	1.94 ± 0.29	0.266	0.244	0.894
*Item 15*	0.88 ± 0.97	0.405	0.337	0.894
*Item 16*	1.90 ± 0.39	0.296	0.267	0.894
*Item 17*	1.71 ± 0.69	0.261	0.208	0.895
*Item 18*	0.99 ± 0.95	0.584	0.531	0.890
*Item 19*	0.97 ± 0.96	0.547	0.489	0.890
*Item 20*	0.62 ± 0.91	0.659	0.615	0.888
*Item 21*	1.50 ± 0.68	0.296	0.246	0.894
*Item 22*	0.57 ± 0.89	0.418	0.357	0.893
*Item 23*	1.37 ± 0.79	0.457	0.405	0.892
*Item 24*	1.12 ± 0.94	0.530	0.473	0.891
*Item 25*	1.60 ± 0.71	0.340	0.288	0.894
*Item 26*	1.01 ± 0.92	0.627	0.579	0.889
*Item 27*	1.50 ± 0.84	0.622	0.579	0.889
*Item 28*	1.51 ± 0.80	0.464	0.412	0.892
*Item 29*	1.22 ± 0.83	0.457	0.402	0.892
*Item 30*	1.03 ± 0.98	0.685	0.641	0.887
*Item 31*	0.99 ± 0.89	0.622	0.575	0.889
*Item 32*	0.54 ± 0.800	0.661	0.622	0.888
*Item 33*	1.10 ± 0.88	0.507	0.452	0.891
*Item 34*	0.57 ± 0.89	0.490	0.434	0.891
*Item 35*	0.88 ± 0.82	0.639	0.598	0.888

*SD* standard deviation

### Convergent and divergent validity

VOC correlated with CRIq-Education (*r* = 0.493, *p* < 0.001), CRIq-Working Activity (*r* = 0.365, *p* = 0.003), CRIq-Leisure time (*r*_*s*_ = 0.421, *p* < 0.001), and CRIq-total (*r* = 0.516, *p* < 0.001) scores. Conversely, divergent validity was confirmed by the absence of correlations between the VOC and BDI-II (*r*_*s*_ =  − 0.034, *p* = 0.811) and STAI-Y (*r* = 0.110, *p* = 0.381).

### Effect of demographic and clinical variables

Multiple regression analysis revealed that a higher level of education was related to better performance on the VOC (*β* = 0.585, *t* = 4.866, *p* < 0.001), whereas no associations emerged with age, sex, EDSS score, and disease duration (Table 3).

**Table 3:** Results for multiple regression analysis with VOC as dependent variable

	Multiple regression analysis			95% confidence limits		Collinearity statistics	
	*β*	*t*	*p*	Lower	Upper	Tolerance	VIF
Age	0.059	0.486	0.629	− 0.175	0.286	0.786	1.273
Sex	0.208	1.927	0.059	− 0.204	10.550	0.995	1.005
Years of schooling	0.585	4.866	** < 0.001**	1.152	2.765	0.801	1.249
EDSS	0.119	0.971	0.336	− 0.873	2.516	0.776	1.288
Disease duration	0.024	0.207	0.837	− 0.007	0.009	0.837	1.195

*VOC* vocabulary knowledge, *EDSS* Expanded Disability Status Scale

### Level of discrimination

**Fig. 1:**
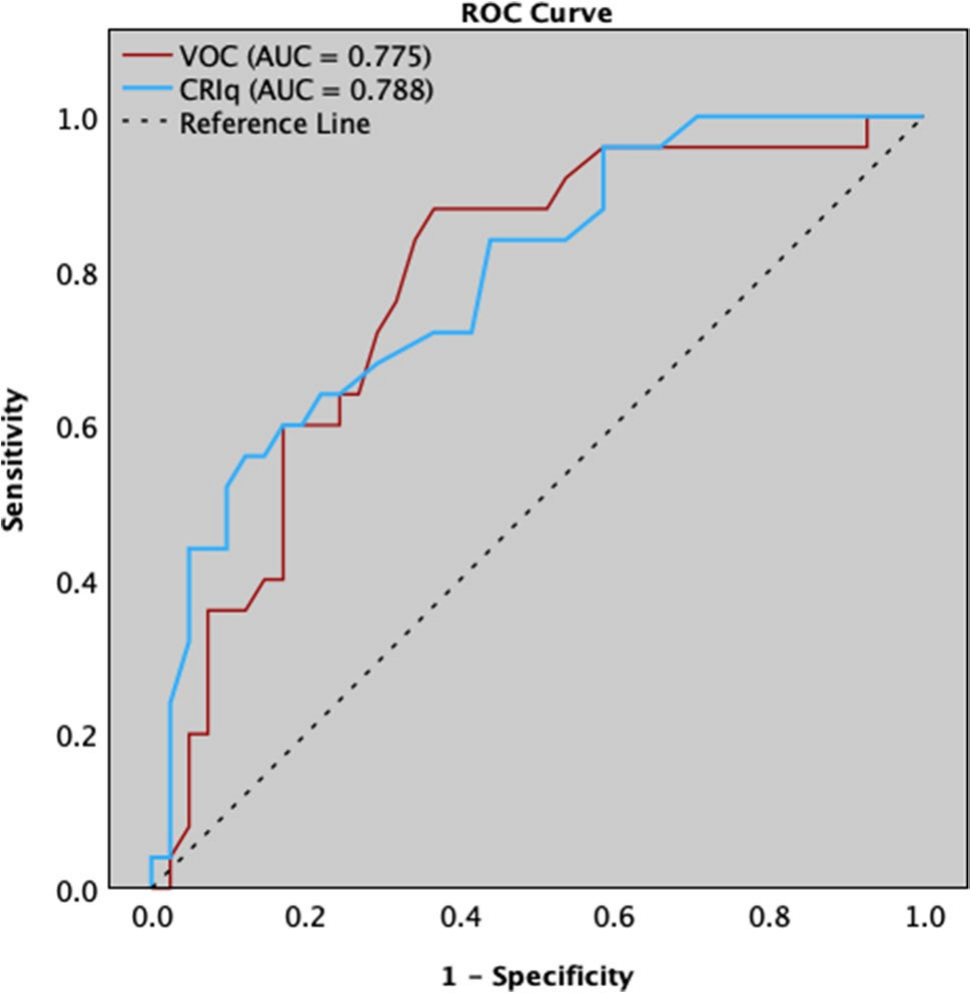
Receiver operating characteristic curves (ROC) of the vocabulary knowledge (VOC) and Cognitive Reserve Index Questionnaire (CRIq) when discriminating people with multiple sclerosis (pwMS) with high and low cognitive reserves, measured using educational level as reference standard

ROC analysis using educational level (years of schooling ≥ 16) as gold standard indicated that VOC demonstrated, at an optimal cutoff of 44.50 (*J* = 0.514), an acceptable level of discrimination of pwMS with high and low CR (AUC = 0.775; *p* < 0.001; SE = 0.059; 95% CI 0.659–0.891) with acceptable intrinsic properties (Se = 0.880; Sp = 0.634) and comparable to the CRIq score (AUC = 0.788; *p* < 0.001; SE = 0.057; 95% CI 0.677–0.899; Se = 0.560; Sp = 0.878) (Fig. 1). Based on the abovementioned cutoff of VOC (< 44, low CR; ≥ 45, high CR), 30 pwMS were classified as having low CR (44.1%) and 38 as having high CR (55.9%).

### Predictors of overall cognition

Multiple regression analysis revealed that higher cognition scores were associated with lower EDSS (*β* =  − 0.394, *t* =  − 3.008, *p* = 0.004) and higher VOC scores (*β* = 0.339, *t* = 2.341, *p* = 0.023). The associations between the overall cognition score and age, sex, education, and disease duration were not significant (Table 4).

**Table 4:** Results for multiple regression analysis with cognitive composite score as dependent variable

	Multiple regression analysis			95% confidence limits		Collinearity statistics	
	*β*	*t*	*p*	Lower	Upper	Tolerance	VIF
Age	− 0.090	− 0.681	0.499	− 0.207	0.102	0.782	1.279
Sex	− 0.155	− 1.291	0.202	− 6.131	1.329	0.946	1.057
Years of schooling	− 0.162	− 1.056	0.296	− 0.986	0.306	0.578	1.730
EDSS	− 0.394	− 3.008	**0.004**	− 2.909	− 0.582	0.795	1.258
Disease duration	− 0.147	− 1.147	0.256	− 0.009	0.002	0.833	1.201
VOC	0.339	2.341	**0.023**	0.030	0.390	0.651	1.537

*EDSS* Expanded Disability Status Scale, *VOC* vocabulary knowledge, *VIF* variance inflation factor

Statistically significant values are reported in bold

## Discussion

The present study aimed to test the feasibility of VOC as a reliable measure of CR in MS. The VOC demonstrated acceptable internal consistency and adequate convergent and divergent validity, as indicated by a significant strong association with the CRIq scale but not with measures assessing depression and anxiety. In addition, this tool showed an acceptable level of discrimination of pwMS with high and low CR and comparable to the CRIq score using educational attainment as reference standard. VOC was associated with educational level but unrelated to age, sex, and clinical features, whereas it emerged as a crucial predictor of cognition, together with EDSS, when controlling for the effect of education and other clinical variables.

Although vocabulary knowledge is frequently used to estimate premorbid intelligence, considering its stability when facing neurologic insult and pathological cognitive decline [34, 35], it may also represent a proxy of CR [36]. Analyzing the capacity of VOC in discriminating between pwMS with high and low CR by ROC analysis, we found that VOC revealed an acceptable level of discrimination accuracy and good intrinsic properties, comparable to those of CRIq, a multi-item interview specifically designed to estimate CR. Indeed, CRIq was demonstrated to be a suitable tool for CR evaluation by combining the most frequently used proxies of CR, such as educational and occupational attainments and leisure activities carried out during an entire adult lifetime [14]. Therefore, the relationship with the CRIq global score, stronger than those with CRIq subscores, proves the convergent validity of the VOC and suggests that it adequately covers the entire spectrum of CR dimensions. Taken together, our findings indirectly confirm that CR relies on static measures, such as years of education and occupational attainment, as well as dynamic proxies, such as literacy and engaging in cognitively stimulating activities, likely to be modified over time [37]. Hence, our work provides further evidence that several factors contribute to the relationship between CR and cognition in clinical and non-clinical populations [38].

Moreover, VOC was not linked to scales assessing neuropsychiatric symptoms such as depression and anxiety. The protective effect of CR in MS is not confined to cognition but may also extend to neuropsychiatric symptoms [24, 39]; however, depression may also impact individuals’ participation in leisure and social activities and moderate the relationship between CR and cognition [40]. Similarly, we found that VOC scores were independent of demographic factors, except for educational level, and MS-related clinical features, such as the level of disability and disease duration. Taken together, these results support the idea that greater lifetime intellectual enrichment is independent of age-related processes and MS-related CNS pathology, thus suggesting that VOC might represent a reliable and standardized proxy of CR in pwMS, since it provides a reliable measure in the presence of neuropsychiatric disturbances.

Furthermore, VOC emerged as a predictor of cognitive status after controlling for educational level, with better performance on BRB-N related to higher VOC and lower EDSS scores. These results not only further confirm findings from previous studies revealing a link between disability and performance on neuropsychological tests in MS [41, 42] but also provide evidence of VOC as a more ecological measure of CR than education in MS. Considering that the MS course and prognosis remain unpredictable, here we propose a cutoff of VOC (44.50) that may help researchers and clinicians to identify pwMS with high and low CR. In fact, reliable CR estimation at the time of diagnosis seems to be a useful clinical predictor of future cognitive decline, driving the identification of patients who might benefit from stricter neuropsychological monitoring and early cognitive interventions [43].

Nevertheless, this study has some limitations. First, the small sample size may limit the generalizability of the results. Second, the cross-sectional nature of the study did not prove the stability of VOC over time; future longitudinal studies should provide evidence of sensitivity to change. Moreover, despite some items demonstrated an unsatisfactory level of discrimination, we decided to retain the original version of the scale. This can be explained by the fact that vocabulary is learned by repeated exposure to words in order to achieve long-term memory retention [44], and thus, the presence of obsolete words may affect individuals’ performance. To this end, a constant update of VOC tests is required to avoid including disused words, thus guaranteeing more reliable estimates of vocabulary knowledge.

In conclusion, we demonstrated the reliability and validity of the VOC as a brief instrument for measuring CR in pwMS. VOC showed good accuracy in discriminating pwMS with low and high CR comparable to specifically designed interviews and emerged as the most influential predictor of cognitive status, independent of demographic and MS clinical features. CR estimation may be crucial to identify pwMS most at risk for future cognitive decline in order to implement cognitive monitoring and timed interventions that can prevent detrimental consequences for patients’ quality of life and their social and professional achievements.

## Data Availability

Datasets associated with the present study are available upon reasonable request of interested researchers.
